# Diagnostic utility of smartphone-integrated gait analysis in the assessment of BPPV

**DOI:** 10.3389/fneur.2025.1728659

**Published:** 2025-12-05

**Authors:** Kasım Durmus, Adem Bora, Baris Sapci, Marwan Khaled Al-Hazzar, Kerem Akti, Melek Kekul Sapci, Emine Elif Altuntas

**Affiliations:** 1 Department of Speech and Language Therapy, Fenerbahce University, Istanbul, Türkiye; 2 Department of Otorhinolaryngology, Sivas Cumhuriyet University, Sivas, Türkiye; 3 Ted Sivas Koleji, Sivas, Türkiye; 4 Department of Healthcare Programme, Vocational School of Health Services, Sivas Cumhuriyet University, Sivas, Türkiye; 5 Department of Otorhinolaryngology, Lokman Hekim Universit, Ankara, Türkiye

**Keywords:** benign paroxysmal positional vertigo, vertigo, gait analysis, mobile applications, smartphone, accelerometer, vestibular disorders

## Abstract

**Background:**

Benign Paroxysmal Positional Vertigo (BPPV) is the most common vestibular disorder causing gait disturbances. Smartphone-based gait analysis has emerged as a portable, cost-effective alternative to laboratory-based assessments.

**Objective:**

To evaluate the diagnostic utility of smartphone-based gait analysis in BPPV patients and its effectiveness in assessing treatment outcomes following canalith repositioning maneuvers.

**Methods:**

This prospective case-control study included 26 patients with posterior canal BPPV and 37 demographically matched healthy controls. Gait analysis was performed using a smartphone application with participants walking 25 meters at a self-selected pace. In the BPPV group, measurements were repeated after Epley maneuver. Patient-reported outcomes were assessed using the Vertigo Symptom Scale (VSS), Vertigo Dizziness Imbalance Symptom Scale (VDI-SS), and Health Related Quality of Life (VDI -HRQoL).

**Results:**

Compared to controls, BPPV patients demonstrated significantly higher number of steps (31.31 ± 6.07 vs. 27.73 ± 6.52, *p* = 0.031), shorter step length (0.54 ± 0.07 m vs. 0.63 ± 0.10 m, *p* < 0.001), and increased vertical center of mass displacement (1.59 ± 0.62 cm vs. 2.75 ± 0.95 cm, *p* < 0.001). Walking speed, cadence, and symmetry parameters showed no significant differences. Following canalith repositioning maneuvers, gait parameters remained unchanged, whereas patient-reported outcome scores improved significantly (VSS: *p* = 0.011, VDI-SS: *p* = 0.002, VDI -HRQoL: *p* = 0.038).

**Conclusion:**

Smartphone-based gait analysis can identify characteristic gait abnormalities in BPPV patients, suggesting its potential as a complementary diagnostic tool. However, it appears less sensitive for detecting early post-treatment biomechanical changes despite symptomatic improvement. Further validation studies with larger samples and extended follow-up periods are warranted.

## 1 Introduction

The vestibular system plays a critical role in maintaining postural stability by perceiving linear and angular accelerations of the head during movement, thereby contributing to the stability of gaze, head, and body while in motion (1). Benign Paroxysmal Positional Vertigo (BPPV) represents the most common vestibular disorder, occurring due to displacement of otoconial debris into the semicircular canals or adherence to the cupula. This displacement triggers transient, intense episodes of vertigo with characteristic head movements, leading to balance impairment and various gait disturbances (2–5).

Traditional BPPV diagnosis relies on medical history, physical examination, and provocation maneuvers such as the Dix–Hallpike and Roll test. While effective, these methods are subjective and lack quantitative measures of gait dysfunction. Conventional gait analysis, performed in specialized laboratories with expensive equipment and trained personnel, has been used to understand the effects of BPPV on postural stability and evaluate treatment outcomes. However, high costs and technical requirements have limited its widespread clinical adoption (3).

Recent advances in smartphone technology have integrated sophisticated accelerometer sensors into mobile devices, enabling measurement of various gait parameters without laboratory settings. Studies have demonstrated the reliability and validity of smartphone-based gait analysis in conditions such as Parkinson’s disease, knee and hip osteoarthritis, hip fractures, and knee prostheses (6–8). Despite these advances, no studies to date have investigated smartphone-based gait analysis specifically in BPPV patients.

This study aimed to address this gap by testing two hypotheses: (1) smartphone-based gait analysis is a useful tool for diagnosing BPPV, and (2) smartphone-based gait analysis is effective for evaluating treatment outcomes in BPPV. We compared gait parameters between BPPV patients and healthy controls and assessed changes in gait metrics and patient-reported outcomes before and after canalith repositioning maneuvers.

## 2 Materials and methods

### 2.1 Study design and participants

This prospective case-control study was conducted at a tertiary university hospital ENT outpatient clinic between March 2023 and June 2023, following approval from the institutional ethics committee (Ethics Committee of Sivas Cumhuriyet University Faculty of Medicine, Date: 22.02.2023, Decision No: 2023-02/08). All participants provided written informed consent.

The study included 26 patients aged 18–75 years diagnosed with posterior canal BPPV based on history, physical examination, and Dix–Hallpike maneuver. Patients attending their second-day follow-up appointment underwent Dix–Hallpike testing following the initial treatment maneuver. Those demonstrating persistent nystagmus or vertigo received repeat treatment with the canalith repositioning maneuver (Epley Maneuver). The control group comprised 37 age- and sex-matched healthy individuals without vestibular complaints.

*Exclusion criteria* included: patients exhibiting continued vertigo after two consecutive canalith repositioning maneuvers, history of lower extremity surgery; neurological or orthopedic disorders affecting gait; developmental hip dysplasia; congenital lower extremity anomalies; lower extremity amputation; body mass index (BMI) ≥40 kg/m^2^; and rheumatologic diseases.

### 2.2 Intervention and assessment timeline

In the BPPV group, the Epley maneuver was performed as the standard canalith repositioning treatment. Gait analysis and questionnaires were administered before treatment and on the second day post-maneuver. The control group underwent a single gait analysis session only.

### 2.3 Smartphone-based gait analysis

Gait analysis was performed using the Gait Analyzer application (version 0.5.3; Control One LLC, NM, USA) installed on an iPhone 13 Pro Max (160.8 × 78.1 × 7.65 mm, 238 g). The methodology followed validated protocols from previous studies (9–12).

**Figure 1:**
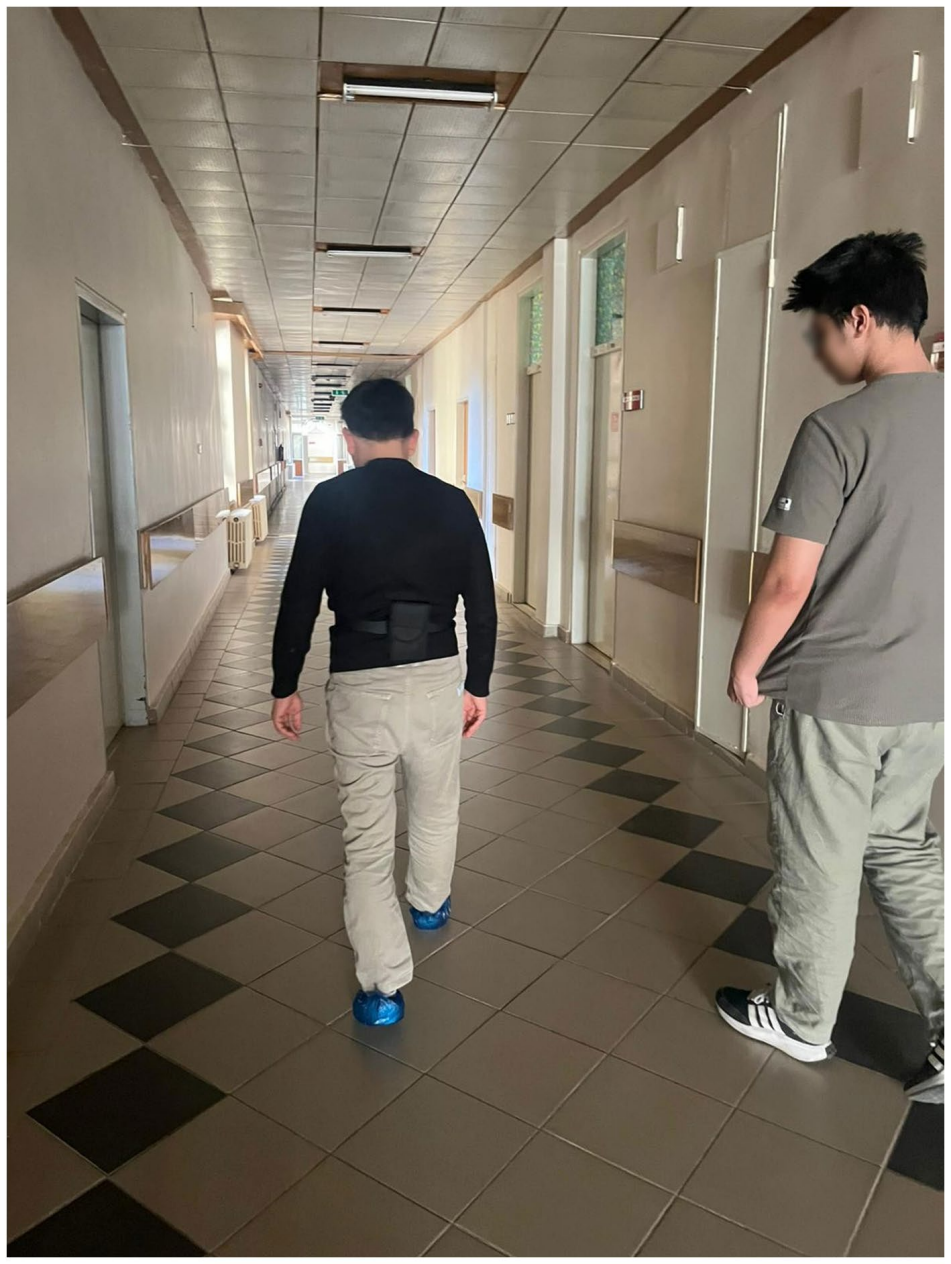
Visual representation of the subject during gait.

*Procedure*: Participants who could walk independently without assistive devices were instructed to walk barefoot at a self-selected pace along a 25-meter walkway. A smartphone was secured horizontally at the level of the third lumbar vertebra (L3) using a belt. An observer followed each participant for safety without influencing walking speed. No falls or adverse events occurred during testing (Figure 1).

**Figure 2:**
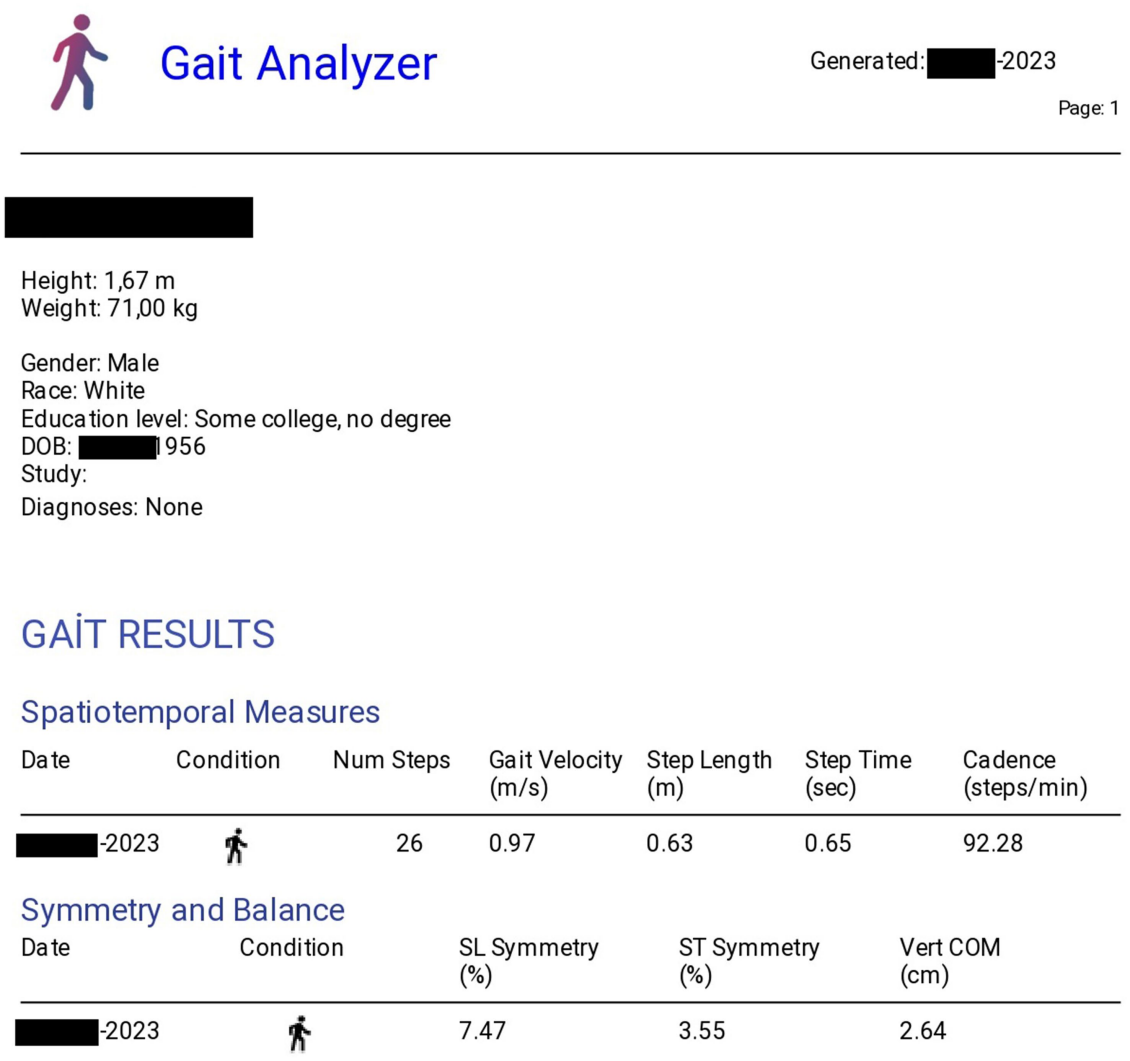
Gait analysis report sample, showing key metrics and visualizations from the analysis software.

*Data acquisition*: The LSM6D accelerometer sensor (STMicroelectronics, Geneva, Switzerland) collected raw acceleration data, which was processed using a low-pass, fourth-order zero-lag Butterworth filter at 20 Hz. Heel-strike events were identified mathematically using proprietary algorithms. The sampling rate used in our study was 50 Hz (Figure 2).

*Measured parameters*:

- Walking velocity (m/s)
- Cadence (steps/min)
- Mean step time (s)
- Mean step length (m)
- Step time symmetry (%)
- Step length symmetry (%)
- Vertical center of mass (CoM) displacement (cm)

*Anthropometric data collected*:

- Height (m)
- Weight (kg)
- Leg length (distance between medial malleolus and anterior superior iliac spine) (m)
- Sex
- Date of birth

### 2.4 Patient-reported outcome measures

Three validated questionnaires were administered to BPPV patients before and after treatment:

1. *Vertigo Symptom Scale (VSS)*: Assesses frequency and severity of vertigo symptoms (maximum score: 75)
2. *Vertigo Dizziness Imbalance Symptom Scale (VDI-SS)*: Evaluates dizziness and imbalance symptoms (maximum score: 70)
3. *Health Related Quality of Life Scale (VDI -HRQoL)*: Measures impact of vertigo on quality of life (maximum score: 110)

These instruments have been validated for use in Turkish BPPV populations (13).

### 2.5 Statistical analysis

Statistical analyses were performed using SPSS version 23.0 (SPSS Inc., Chicago, IL, USA). Descriptive statistics included mean, standard deviation, median, and range. Normality was assessed using Kolmogorov–Smirnov or Shapiro–Wilk tests. Parametric tests were applied to normally distributed data, while nonparametric tests were used otherwise. Categorical variables were analyzed using chi-square tests. Dependent and independent group comparisons utilized appropriate *t*-tests or Mann–Whitney U tests. Statistical significance was set at *p* < 0.05.

Power analysis was performed using G*Power software with reference (6) as the basis for calculations. Given an effect size of 0.98, a significance level (*α*) of 0.05, and desired power (1-*β*) of 0.95, the adequate sample size was calculated as 24 patients per group. When the study was conducted with 24 patients per group, the actual power achieved was 0.96.

## 3 Results

### 3.1 Demographic characteristics

No statistically significant differences were observed between BPPV patients and controls regarding age, sex, or BMI (Table 1).

**Table 1:** Demographic characteristics of study groups.

Parameter	BPPV group (*n* = 26)	Control group (*n* = 37)	*p*-value
Gender (Female/Male)	17/9	16/21	0.083
Age (years)	53.61 ± 15.57	48.91 ± 10.99	0.165
BMI (kg/m^2^)	27.49 ± 4.36	25.71 ± 3.56	0.079

BMI, body mass index.

### 3.2 Comparison of gait parameters between BPPV and control groups

BPPV patients demonstrated significantly more steps compared to controls (*p* = 0.031), while step length and vertical CoM displacement were significantly reduced (*p* < 0.001 for both). No significant differences were found in walking speed, cadence, step time, or symmetry parameters (Table 2).

**Table 2:** Comparison of gait parameters between bppv and control groups.

Parameter	BPPV group (*n* = 26)	Control group (*n* = 37)	*p*-value
Number of steps	31.31 ± 6.07	27.73 ± 6.52	0.031*
Gait velocity (m/s)	0.90 ± 0.17	1.01 ± 0.26	0.067
Step length (m)	0.54 ± 0.07	0.63 ± 0.10	<0.001*
Step time (sec)	0.63 ± 0.14	0.67 ± 0.15	0.374
Cadence (steps/min)	99.51 ± 16.79	96.17 ± 20.08	0.490
Step length symmetry (%)	17.74 ± 8.91	16.04 ± 9.24	0.542
Step time symmetry (%)	20.49 ± 21.43	23.97 ± 18.47	0.494
Vertical CoM displacement (cm)	1.59 ± 0.62	2.75 ± 0.95	<0.001*

*CoM, center of mass; Statistically significant.

### 3.3 Effect of Canalith repositioning maneuver on gait parameters

When comparing pre- and post-maneuver gait analyses in BPPV patients, no statistically significant changes were observed in any gait parameters (Table 3).

**Table 3:** Gait parameters before and after canalith repositioning maneuver in BPPV patients.

Parameter	Before maneuver (*n* = 26)	After maneuver (*n* = 26)	*p*-value
Number of steps	31.31 ± 6.07	29.12 ± 4.89	0.158
Gait velocity (m/s)	0.90 ± 0.17	0.93 ± 0.21	0.545
Step length (m)	0.54 ± 0.07	0.56 ± 0.10	0.377
Step time (sec)	0.63 ± 0.14	0.63 ± 0.11	0.983
Cadence (steps/min)	99.51 ± 16.79	97.53 ± 12.89	0.634
Step length symmetry (%)	17.47 ± 8.91	16.43 ± 8.04	0.660
Step time symmetry (%)	20.49 ± 21.43	18.00 ± 17.60	0.648
Vertical CoM displacement (cm)	1.59 ± 0.62	2.05 ± 1.12	0.070

CoM, center of mass.

### 3.4 Patient-reported outcomes

In contrast to objective gait parameters, all patient-reported outcome measures demonstrated significant improvement after treatment. VSS, VDI-SS, and VDI -HRQoL scores all showed statistically significant reductions, indicating symptomatic improvement (Table 4).

**Table 4:** patient-reported outcomes before and after canalith repositioning maneuver.

Questionnaire (maximum score)	Before maneuver (*n* = 26)	After maneuver (*n* = 26)	*p*-value
VSS (75)	25.23 ± 17.24	14.46 ± 11.74	0.011*
VDI-SS (70)	33.85 ± 17.01	18.58 ± 15.94	0.002*
VDI -HRQoL (110)	44.15 ± 27.73	28.54 ± 25.05	0.038*

*VSS, Vertigo Symptom Scale; VDI-SS, Vertigo Dizziness Imbalance Symptom Scale; VDI –HRQoL, Vertigo Dizziness Imbalance Health Related Quality of Life Scale; Statistically significant.

## 4 Discussion

This study demonstrates that smartphone-based gait analysis can identify characteristic biomechanical abnormalities in BPPV patients compared to healthy controls. Our most significant finding is that while walking speed was not significantly different between groups, BPPV patients exhibited significantly more steps with reduced step length and decreased vertical CoM displacement. These findings suggest that BPPV patients adopt compensatory gait strategies to maintain dynamic balance by taking shorter, more frequent steps and minimizing trunk sway.

### 4.1 Gait abnormalities in BPPV

Our results align with previous laboratory-based studies. Kara et al. (14) used wearable sensors to demonstrate similar gait alterations in BPPV patients. Cohen-Shwartz et al. (3) reported shortened step length and reduced gait speed in BPPV patients compared to controls. Zhang et al. (1) demonstrated decreased step length, gait speed, and cadence, along with impaired gait stability. The consistency between our smartphone-based findings and laboratory studies supports the validity of mobile technology for BPPV assessment.

The observed gait pattern more frequent, shorter steps with reduced vertical CoM displacement likely represents a compensatory mechanism to enhance stability. By taking shorter steps and minimizing vertical movement, patients may reduce the destabilizing effects of vestibular dysfunction during locomotion. This adaptation reflects the brain’s attempt to prioritize balance over efficiency in the presence of vertigo-induced instability.

### 4.2 Smartphone technology as a diagnostic tool

Traditional gait analysis requires expensive equipment, dedicated laboratory space, and trained personnel, limiting its clinical accessibility (15). The integration of accelerometer sensors into smartphones has created opportunities for portable, cost-effective gait assessment. Previous studies have validated smartphone-based gait analysis against laboratory standards, demonstrating high reliability and validity (9, 16, 17).

The Gait Analyzer application used in this study enables clinicians to obtain objective gait measurements without specialized equipment or technical expertise. Unlike other systems requiring data transfer and complex processing, this application provides immediate, user-friendly analysis of spatiotemporal gait parameters (18, 19). This accessibility could facilitate early identification of vestibular disorders in primary care and outpatient settings.

Beyond accelerometer data, future developments may integrate smartphone camera technology for simultaneous video analysis, potentially providing even more comprehensive assessment tools. Such multimodal approaches could enable clinicians to obtain objective, quantifiable measurements of gait dysfunction without laboratory resources, advancing the diagnosis and monitoring of BPPV and other vestibular disorders.

### 4.3 Post-treatment assessment and gait recovery

For posttreatment assessment, we utilized the VSS, introduced by Yardley et al. (20). This scale was developed to provide a self-reported measure of vertigo severity that would not be confounded by anxiety-related symptoms, thereby enabling a clearer assessment of the independent contributions of vertigo and anxiety to patient-perceived handicap and distress. Subsequently, in 1999, Prieto et al. (21) designed the VDI questionnaire as an outcome measure for clinical trials, with the aim of monitoring treatment progress and comparing patient groups experiencing these symptoms. Subsequent validation studies have demonstrated that the VDI is a reliable, valid, and responsive instrument for evaluating vertigo, dizziness, and imbalance.

A notable finding of our study is the discordance between objective gait parameters and subjective symptom improvement following canalith repositioning maneuvers. While VSS, VDI-SS, and VDI -HRQoL scores showed significant improvement, gait parameters remained unchanged at the early post-treatment assessment. This suggests that symptomatic relief precedes biomechanical recovery, or that smartphone-based gait analysis lacks sufficient sensitivity to detect subtle early improvements.

In our study, we conducted post-treatment assessments on the second day following the canalith repositioning maneuver. However, the optimal timing for post-treatment evaluation remains controversial in the literature. While some authors have reported that symptom resolution may begin as early as two hours after the Epley maneuver (22), others have documented that complete recovery can extend up to one month (23). Further studies examining different time intervals for post-treatment assessment are warranted.

Our findings are consistent with recent meta-analytic data. Pauwels et al. (24) reported that while walking speed on flat surfaces improved significantly after canalith repositioning, improvements in more challenging tasks like tandem walking or walking with head movements were inconsistent. This suggests that simple gait parameters may not capture the full spectrum of vestibular recovery, particularly in the early post-treatment period.

Lim et al. (25) evaluated dynamic gait performance before and after treatment in BPPV patients, reporting increased tandem walking ability and walking speed in some but not all patients. This partial improvement pattern aligns with our observation of unchanged gait parameters despite symptomatic improvement. These findings suggest that gait recovery in BPPV may be a gradual process, with restoration of basic walking function preceding improvement in more demanding locomotor tasks.

Several mechanisms may explain this dissociation between symptoms and objective gait measures:

1. *Vestibular compensation*: While the canalith repositioning maneuver resolves the mechanical cause of vertigo, central vestibular compensation may require additional time to fully restore normal gait mechanics.
2. *Measurement sensitivity*: The spatiotemporal parameters measured during simple overground walking may not be sufficiently sensitive to detect subtle changes in gait quality or stability that occur early in recovery.
3. *Task difficulty*: Walking at a self-selected pace on a flat surface represents a relatively simple motor task. More challenging conditions—such as dual-task walking, tandem gait, or walking with head movements—might reveal deficits and improvements not apparent in simple walking.
4. *Psychological factors*: Rapid symptomatic improvement may reflect resolution of anxiety and vertigo symptoms, while biomechanical patterns established during symptomatic periods may persist due to motor learning or caution.

### 4.4 Validity of patient-reported outcomes

The significant improvement in VSS, VDI-SS, and VDI -HRQoL scores after treatment confirms the effectiveness of canalith repositioning maneuvers and validates these instruments for assessing treatment response. Yanik et al. (13) previously demonstrated that these questionnaires are reliable and valid tools for Turkish BPPV populations. Our results further support their utility in clinical practice and research.

The discrepancy between objective and subjective measures highlights the importance of using multiple assessment modalities. While patient-reported outcomes capture the lived experience of illness and treatment benefit, objective gait analysis may provide complementary information about residual biomechanical dysfunction requiring additional intervention or extended recovery time.

### 4.5 Clinical implications

Our findings have several clinical implications:

1. *Diagnostic adjunct*: Smartphone-based gait analysis could serve as an objective, quantifiable adjunct to traditional BPPV diagnosis, particularly useful for:

- Documenting baseline dysfunction.
- Differentiating BPPV from other causes of dizziness.
- Identifying patients with significant gait impairment who may benefit from falls prevention strategies.

- *Screening tool*: The portability and low cost of smartphone-based assessment make it suitable for screening in primary care settings, potentially facilitating earlier diagnosis and treatment.
- *Monitoring recovery*: While not sensitive to early post-treatment changes, smartphone-based gait analysis may be useful for monitoring longer-term recovery and identifying patients with persistent gait dysfunction despite symptom resolution.
- *Research applications*: Standardized smartphone-based measurements could facilitate multi-center research by providing consistent, objective outcome measures across different institutions.

### 4.6 Limitations

Several limitations should be considered when interpreting our results:

1. *Sample size*: The relatively small number of participants (26 BPPV patients, 37 controls) may have limited statistical power to detect small differences or changes in some gait parameters.
2. *Single device*: Using a single smartphone model ensured measurement consistency but prevented assessment of inter-device reliability. Different smartphones have varying accelerometer specifications, which may affect measurement accuracy and comparability.
3. *Follow-up duration*: The short post-treatment follow-up (second day after maneuver) may have been insufficient to observe biomechanical improvements that require longer adaptation periods. Extended follow-up at 1 week, 1 month, and 3 months post-treatment would provide more comprehensive understanding of gait recovery trajectories.
4. *Simple walking task*: Assessment during simple overground walking at self-selected speed may not reveal deficits apparent under more challenging conditions such as:

- Tandem (heel-to-toe) walking.
- Walking with head movements.
- Dual-task walking (walking while performing cognitive tasks).
- Walking on uneven surfaces.
- Walking at different speeds (slow and fast).

- *Single canal involvement*: Our study included only posterior canal BPPV. Patients with horizontal or anterior canal involvement may exhibit different gait patterns.
- *Application validation*: While the Gait Analyzer application has been validated in other populations, specific validation in BPPV patients would strengthen our findings.

### 4.7 Future directions

Future research should address these limitations and expand our understanding of smartphone-based gait analysis in BPPV:

1. *Multi-center validation*: Large-scale studies involving multiple institutions and diverse patient populations would establish generalizability and reference values.
2. *Device comparison*: Systematic comparison of different smartphone models and operating systems would determine inter-device reliability and establish whether device-specific corrections are necessary.
3. *Extended follow-up*: Longitudinal studies with assessments at multiple time points (1 week, 1 month, 3 months, 6 months post-treatment) would characterize the full trajectory of gait recovery.
4. *Challenging conditions*: Incorporation of more demanding walking tasks (tandem gait, head movement walking, dual-task conditions) may reveal sensitivity to detect post-treatment improvements.
5. *Machine learning applications*: Advanced analytical approaches using machine learning algorithms could identify subtle patterns in accelerometer data that discriminate BPPV from other vestibular disorders or predict treatment outcomes.
6. *Integration with other sensors*: Combining accelerometer data with gyroscope, magnetometer, and camera data could provide more comprehensive assessment of gait and balance function.
7. *Falls prediction*: Investigation of whether specific gait parameters identify BPPV patients at high risk for falls could inform targeted prevention strategies.
8. *Cost-effectiveness analysis*: Formal evaluation of the cost-effectiveness of smartphone-based assessment compared to traditional methods would support clinical implementation decisions.

## 5 Conclusion

This study provides initial evidence that smartphone-based gait analysis can identify characteristic gait abnormalities in BPPV patients, particularly increased step count, reduced step length, and decreased vertical CoM displacement. These findings support the potential utility of mobile technology as a complementary diagnostic tool for BPPV, offering objective, quantifiable measurements that could enhance traditional clinical assessment.

However, smartphone-based gait analysis demonstrated limited sensitivity for detecting early post-treatment biomechanical changes, despite significant symptomatic improvement as measured by patient-reported outcomes. This suggests that while the technology shows promise for diagnosis and screening, its role in treatment monitoring may be limited to longer-term follow-up or may require more challenging assessment conditions.

The widespread availability of smartphones and the development of validated applications create unprecedented opportunities for accessible, objective assessment of vestibular disorders. As mobile health technologies continue to advance, early detection and monitoring of common conditions like BPPV may become feasible in diverse clinical settings. Further validation studies involving larger populations, multiple devices, extended follow-up periods, and more challenging walking tasks are warranted to fully establish the clinical utility of smartphone-based gait analysis in BPPV management.

## Data Availability

The raw data supporting the conclusions of this article will be made available by the authors, without undue reservation.
