# Correction: Diagnostic utility of smartphone-integrated gait analysis in the assessment of BPPV

**DOI:** 10.3389/fneur.2025.1772096

**Published:** 2026-01-12

**Authors:** Kasim Durmus, Adem Bora, Baris Sapci, Marwan Khaled Al-Hazzar, Kerem Akti, Melek Kekul Sapci, Emine Elif Altuntas

**Affiliations:** 1 Department of Speech and Language Therapy, Fenerbahce University, Istanbul, Türkiye; 2 Department of Otorhinolaryngology, Sivas Cumhuriyet University, Sivas, Türkiye; 3 Ted Sivas Koleji, Sivas, Türkiye; 4 Department of Healthcare Programme, Vocational School of Health Services, Sivas Cumhuriyet University, Sivas, Türkiye; 5 Department of Otorhinolaryngology, Lokman Hekim Universit, Ankara, Türkiye

**Keywords:** benign paroxysmal positional vertigo, vertigo, gait analysis, mobile applications, smartphone, accelerometer, vestibular disorders

In the published article, affiliation 1 “Department of Speech and Language Therapy, Fenerbahce University, Istanbul, Türkiye” was erroneously written as “Medicana International Izmir Hospital, Izmir, Türkiye”.

The original version of this article has been updated.

